# Pharmacokinetics and Tissue Distribution of Combined Triptolide and Paeoniflorin Regimen for Percutaneous Administration in Rats Assessed by Liquid Chromatography-Tandem Mass Spectrometry

**DOI:** 10.1155/2021/8864273

**Published:** 2021-07-08

**Authors:** Yong-mei Guan, Qian Shen, Liang-fei He, Li-mei Chen, Zhenzhong Zang, Lili Liu, Wei-feng Zhu, Chen Li-hua, Hong-ning Liu

**Affiliations:** ^1^Jiangxi University of Chinese Medicine, Nanchang, Jiangxi, China; ^2^The Affiliated Hospital of Jiangxi University of TCM, Nanchang, Jiangxi, China

## Abstract

Triptolide (TP) has shown potential in rheumatoid arthritis (RA) treatment, but the narrow therapeutic window limits its clinical application. In clinical practice, the compatibility of *Tripterygium wilfordii* and *Paeonia lactiflora* is often used to attenuate the toxicity of TP, but its compatibility mechanism has not been fully elucidated. The aim of this study was to investigate the pharmacokinetics and tissue distribution of a combined regimen of TP and paeoniflorin (PF) after transdermal administration in male and female Sprague Dawley (SD) rats via a rapid and sensitive liquid chromatography-tandem mass spectrometry (LC-MS/MS) method. The results showed that after percutaneous administration of TP and PF, there was no significant difference in AUC _(0-*t*)_ (area under the curve) of TP, the peak concentration decreased by 58.17%, and the peak time was delayed. The AUC _(0-t)_ of PF increased significantly (*P* < 0.01), the peak-reaching concentration and AUC _(0-∞)_ increased, and the half-life and average retention time were shortened, indicating that TP absorption in rats may be delayed. After percutaneous administration of TP and PF, the content of TP in the heart, liver, spleen, lungs, and kidneys of male rats significantly decreased at 2 h (*P* < 0.05) and the drug concentration in the liver tissues significantly decreased at 2 h, 4 h, and 8 h (*P* < 0.05). The TP content in the spleen of female rats significantly decreased at 2 h and 4 h (*P* < 0.05) and also decreased in other tissues, but not significantly. After percutaneous administration of TP and PF, the PF content in the heart, liver, spleen, lungs, and kidneys of male and female rats had no significant difference. However, after percutaneous administration of TP and PF, the TP concentration in the skin increased, suggesting that the amount of TP retained in the skin increased, thereby reducing its content in blood and tissues, producing a reduction in toxicity effect.

## 1. Introduction

Triptolide (TP) is an epoxy-xylene lactone compound. The common ingredient of *Tripterygium wilfordii* hook, TP, is responsible for anti-inflammatory, antitumor, antifertility, and immunomodulatory effects [1–3]. Clinically, TP has a clear curative effect on rheumatoid arthritis (RA), but its narrow therapeutic window and strong liver and kidney toxicity limit its safe clinical application [4]. Our research group used data mining technology to summarize and analyze the use of *Tripterygium wilfordii* in the treatment of rheumatoid arthritis [5–7]. It was found that *Tripterygium wilfordii* was often administered in combination with *Paeonia lactiflora*, and it was reported that the combination of the two could enhance efficacy and reduce toxicity [8–12]; however, these combinations were mostly orally administered. The mechanism of transdermal administration of *Tripterygium wilfordii* combined with *Paeonia lactiflora* is unclear.

The transdermal drug delivery system (TDDS) refers to a system in which drugs are administered on the surface of the skin, pass through each layer of the skin at a constant rate, enter systemic circulation, and produce systemic or local therapeutic effects [13]. The preparation of TP as a topical preparation for transdermal administration is beneficial to reduce the first-pass effects and gastrointestinal tract toxicity and enable the drug to enter the systemic circulation in a small, continuous amount so as to reduce toxicity. It was found that transdermal administration of *Tripterygium wilfordii* can maintain a stable blood drug concentration [14]. Additionally, *Tripterygium wilfordii* has been prepared into microemulsion gel, and pharmacodynamic experiments conducted on CIA model rats found that the topical preparation of *Tripterygium wilfordii* had a curative effect [15, 16]. However, long-term use alone still produces more serious toxicity to the organism.

Therefore, in this study, modern techniques and analytical methods were used to further study the pharmacokinetics and tissue distribution of TP and PF (the main active component of *Paeonia lactiflora*) after transdermal administration in order to explain the mechanism of toxicity reduction caused by combining the two drugs.

## 2. Materials and Methods

### 2.1. Materials

Triptolide (Chengdu Puffer Biotechnology Co., Ltd., Chengdu, China, purity ≥98%), paeoniflorin (Chengdu Puffer Biotechnology Co., Ltd., purity ≥98%), AB Triple Quadrupole 4500 (AB SCIEX, USA), AB Triple Quadrupole 5500 (AB SCIEX, USA), and Agilent InfinityLab Poroshell 120 SB-C18 (2.1 × 50 mm, 2.7 *μ*m) were used.

### 2.2. Animals

Sprague Dawley (SD) rats weighing 220–250 g were obtained from Hunan Slake Jingda Experimental Animal Co., Ltd., and acclimated in the laboratory for seven days. Prior to the experiments, rats were housed with free access to food and water on a 12 h light-dark cycle at ambient temperature (22–24°C) and roughly 50% relative humidity. All animal procedures were approved by the Ethics Committee of the Experimental Animal Science and Technology Center of Jiangxi University of Traditional Chinese Medicine (No. JZLLSC2019-0149).

### 2.3. Preparation of TP Cream

Oil phase contained isopropyl myristate (10%), glyceryl monostearate (14%), and Span-60 (2%); aqueous phase contained Tween-80 (2%), Garandam (0.2%), and water (68.8%); drug: 0.01% TP is dissolved in 3% anhydrous ethanol. First, the oil phase was preheated in a water bath at 80°C for 30 min. Next, the drug was poured into the oil phase and stirred evenly, after which the water phase was quickly poured into the oil phase. The mixture was sheared at high speed of 10,000 r·min^−1^ for 2 min, stirred, and cooled in an ice-water bath to obtain TP cream.

### 2.4. Preparation of PF Cream

Oil phase contained isopropyl myristate (10%), glyceryl monostearate (14%), and Span-60 (2%); aqueous phase contained Tween-80 (2%), Garandam (0.2%), water (58.2%), and PF (10%). The oil phase was preheated in a water bath at 80°C for 30 min, after which the water phase was quickly poured into the oil phase. The mixture was sheared at high speed of 10,000 r·min^−1^ for 2 min, stirred, and cooled in an ice-water bath to obtain PF Cream.

### 2.5. Preparation of the Combination of TP and PF Cream (TP-PF)

Oil phase contained isopropyl myristate (10%), glyceryl monostearate (14%), and Span-60 (2%); aqueous phase contained Tween-80 (2%), paeoniflorin (10%), and Galandan (0.2%), water (58.2%); drug: 0.01% TP is dissolved in 3% anhydrous ethanol. First, the oil phase was preheated in a water bath at 80°C for 30 min. Next, the drug was poured into the oil phase and stirred evenly, after which the water phase was quickly poured into the oil phase. The mixture was sheared at high speed of 10,000 r·min^−1^ for 2 min, stirred, and cooled in an ice-water bath to obtain TP-PF cream.

### 2.6. Drug Administration and Sampling

For the pharmacokinetic study, rats were divided into three groups (six males and six females per group): the TP, PF, and TP-PF groups. Before the experiment, the rats were fasted for 16 h but allowed water. The back hair (5 cm × 4 cm) of the rats was removed. TP (1 mg·kg^−1^·D^−1^), PF (1 g·kg^−1^·D^−1^), and TP-PF creams were uniformly applied in the area from which the back hair was removed. The surface of the treatment area was covered with polyethylene and fixed with a medical adhesive cloth. Then, 0.25 mL blood samples were collected in heparinized Eppendorf tube via the posterior orbital venous plexus before dosing and subsequently at 0.083 h, 0.25 h, 0.5 h, 1 h, 2 h, 3 h, 4 h, 6 h, 8 h, 12 h, and 24 h. After centrifuging at 4000 r·min^−1^ for 10 min, the plasma samples were obtained and frozen at −20°C until analysis. For the tissue distribution study, three groups of rats (six males and six females per group) underwent drug administration in the same manner. Heart, liver, spleen, lungs, kidneys, skin, ovaries, and testes were removed at 2 h, 4 h, and 8 h after dosing. Tissue samples were weighed and rinsed with physiological saline solution; then, the samples were blotted using filter paper and stored at −20°C until analysis.

### 2.7. Plasma and Tissues Samples Processing

In the analysis of biological samples in vivo, due to the low drug concentration, it is easy to produce large errors. Therefore, we added internal standards to reduce the errors in the sample processing process. In this article, we chose carbamazepine as the internal standard because the retention times of carbamazepine and triptolide and paeoniflorin were similar and the peak shape was better under the chromatographic conditions of the two, and they have passed the methodological verification.

To a 100 *μ*L aliquot of plasma sample, 10 *μ*L mixed standard, 10 *μ*L internal standard (IS) (5 ng/mL) working solution, and 20 *μ*L ammonia water were added in turn. All tissues were homogenized in triple normal saline. To a 100 *μ*L of tissues homogenate sample, 10 *μ*L mixed standard, 10 *μ*L IS (5 ng/mL) working solution, and 20 *μ*L ammonia water were added in turn. Plasma and tissue samples were then vortex-mixed for 30 s and extracted with 0.8 mL ethyl acetate by vortex mixing for 3 min. After centrifugation at 12,000 r·min^−1^ for 3 min, 0.78 mL upper organic layer was transferred. Next, 10 *μ*L ammonia and 400 *μ*L ethyl acetate were added to the lower layer and vortex-mixed for 2 min. After centrifugation at 12,000 r·min^−1^ for 3 min, 400 *μ*L upper organic layer was transferred, and the two upper organic layers were combined. The combined upper organic layer was evaporated to dryness at 50°C under a gentle stream of nitrogen. The residue was reconstituted in 100 *μ*L methanol, vortex-mixed for 1 min, and centrifuged at 16,000 r·min^−1^ for 10 min. Finally, a 50 *μ*L aliquot was injected into the liquid chromatography-tandem mass spectrometry (LC-MS/MS) system for analysis.

### 2.8. LC-MS/MS Conditions and Method Validation

The chromatographic conditions for TP were as follows: solvent A was 0.1% formic acid (V/V) in water, and solvent B was a gradient elution with acetonitrile. The elution conditions were as follows: 0–1 min, 65% A; 1–1.5 min, 65–52% A; 1.5–2.5 min, 52% A; 2.5–2.6 min, 52%–10% A; 2.6–3.5 min, 10% A; 3.5–4.1 min, 10%–65% A; 4.1–5 min, 65% A. The flow rate was 0.4 mL/min, and the column temperature was 40°C. The TP was detected in positive ion mode. An electrospray ion source was used, and the ion source temperature was 550°C, ionized voltage was 5500 V, curtain gas was at 35 psi, and impact gas was at 7 psi. Auxiliary gas 1 (GS1) was 55 psi, auxiliary gas 2 (GS2) was 50 psi, inlet voltage (EP) was 10 V, and outlet voltage (CXP) was 14 V. The quantification ion pairs of TP were 361.2/144.8. Additionally, the quantification ion pairs of internal carbamazepine were 236.8/194.0.

The chromatographic conditions for PF were as follows: solvent A was 0.1% formic acid (V/V) in water, and solvent B was a gradient elution with acetonitrile. The elution conditions were as follows: 0–1 min, 90–65% A; 1–1.5 min, 65–44% A; 1.5–3 min, 44% A; 3–3.1 min, 44%–90% A; 3.1–5 min, 90% A. The flow rate was 0.35 mL/min, and the column temperature was 40°C. The TP was detected in positive ion mode. An electrospray ion source was used, and the ion source temperature was 550°C, ionized voltage was 5500 V, curtain gas was at 35 psi, and impact gas was at 9 psi. Auxiliary gas 1 (GS1) was 50 psi; auxiliary gas 2 (GS2) was 50 psi; inlet voltage (EP) was 10 V; outlet voltage (CXP) was 6 V. The quantification ion pairs of PF were 498.2/179.0. Also, the quantification ion pairs of internal carbamazepine were 236.8/194.0.

### 2.9. Statistical Analysis

All the data were expressed as mean ± standard deviation (SD), and statistical analyses were performed by SPSS 17.0. The pharmacokinetic data were fitted by DAS 2.0 software and then tested by SPSS 17.0. Pharmacokinetic parameters and analyte levels in the male and female rats were assessed by one-way ANOVA followed by a least-significant-difference test. *P* < 0.05 was considered to indicate statistical significance, and *P* < 0.01 was deemed to indicate a highly significant difference.

## 3. Results

### 3.1. Specificity

Specificity was evaluated by comparing the chromatogram of blank plasma with that of blank plasma spiked with the mixed standard of TP (400 ng/mL) and PF (90,000 ng/mL), as previously described and that of plasma obtained from rats after administration of TP and PF after treatment for 30 min, respectively. For the pharmacokinetic study, the results showed that TP, PF, and IS were eluted, and no detectable interfering peaks were found (Figure S1). For the tissue distribution study, the result showed that TP, PF, and IS were eluted, and no detectable interfering peaks were found (Figure S2).

### 3.2. Linearity

Linearity of the developed analytical method was investigated by analyzing the matrix-matched construction via an internal standard approach, using mixed probe drugs at a series of concentrations. TP was used at the following concentrations: 50, 100, 250, 500, 1250, 2500, and 5000 ng/mL at 25°C. PF was used at concentrations of 1, 5, 10, 100, 250, 500, and 1000 *μ*g/mL. IS was used at concentrations of 5 ng/mL. Taking the ratio of the target component to the peak area of IS as the longitudinal coordinate and the concentration as the transverse coordinate, linear regression was performed. The standard curve was drawn with the reciprocal of the concentration as the weighted coefficient. For the pharmacokinetic study, the calibration curves constructed by plotting the peak area ratios of TP and PF to IS versus the nominal concentrations in the standard biological samples using linear regression analysis are listed in Table 1. The calibrations were linear over a certain range in all matrices, and the correlation coefficients (*r*^2^) of TP and PF were 0.9949 and 0.9928, respectively. For the tissue distribution study, the linear regression analysis is listed in Tables 2 and 3. The correlation coefficients (*r*^2^) of TP in the heart, liver, spleen, lungs, kidneys, skin, ovaries, and testes were 0.9979, 0.9941, 0.9991, 0.999, 0.9981, 0.9985, 0.9949, and 0.998, respectively. The correlation coefficients (*r*^2^) of PF in the heart, liver, spleen, lungs, kidneys, skin, ovaries, and testes were 0.998, 0.996, 0.997, 0.997, 0.993, 0.995, 0.998, and 0.993, respectively.

### 3.3. Accuracy and Precision

In total, six replicate analyses of the quality control (QC) samples (TP, PF, and IS) were prepared at three different concentrations (the concentrations of TP were 150 ng/mL, 500 ng/mL, and 4000 ng/mL, and the concentrations of PF and IS were 3 *μ*g/mL, 100 *μ*g/mL, and 900 *μ*g/mL) on the same day to ensure interday accuracy and precision. The present study estimated the intraday precision by analyzing six replicate QC samples on three consecutive days. The relative standard deviation (RSD) was used to assess precision, and accuracy was defined as the percent ratios of the calculated concentration to the nominal concentrations. For the pharmacokinetic study, the intra- and interday precision and accuracy for QC samples are listed in Table 4. For the tissue distribution study, the intra- and interday precision and accuracy for QC samples are listed in Tables 5 and 6. It was demonstrated that the results were all acceptable (RSD, <15%).

### 3.4. Recovery Rate

The ratio of the actual measured concentration to the marked concentration was used to determine the recovery rate of the analytical method. For the pharmacokinetic study, the recovery rate of TP was acceptable, the RSD of TP and PF was less than 15%, and there was no obvious matrix effect (Table S1). For the tissue distribution study, the recovery rate of TP was higher in the homogenate of different tissues. The RSD of TP and PF was less than 15%, and there was no obvious matrix effect (Tables S2 and S3).

### 3.5. Stability

QC samples at three concentrations (low, medium, and high) were used to assess the freeze-thaw cycle stabilities of the mixed TP, PF, and IS. All QC samples were stored at −80°C and subjected to three freeze-thaw cycles. Each cycle lasted for 20 h, and the concentrations were determined using LC-MS. For the pharmacokinetic study, the results of stability studies showed that the RSD was less than 15%, indicating that the sample has good stability under various conditions (Tables S4 and S5). For the tissue distribution study, the results of stability studies showed that the stability of the sample was good under various conditions (Tables S6 and S7).

### 3.6. Pharmacokinetics

**Figure 1:**
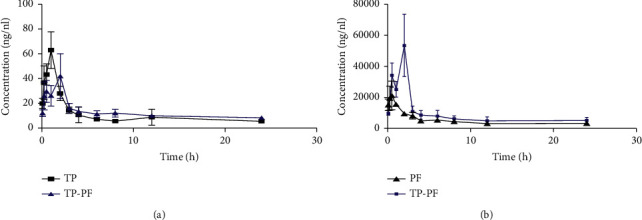
Blood concentration-time curve before and after compatibility. (a) TP. (b) PF.

The plasma concentration-time curves of TP and PF in each group of creams after transdermal administration are depicted in Figure 1. The corresponding pharmacokinetic parameters generated by fitting plasma concentration profiles to a noncompartmental model are listed in Table 7. The results showed that both TP and PF can be rapidly absorbed in the body and slowly eliminated after reaching a certain level, and both compartment models were two-compartment models. After transdermal administration of the combination of TP and PF, the AUC _(0-∞)_ of TP in the combination group increased (*P* < 0.01), *C*_max_ was decreased by 58.17%, the AUC _(0-*t*)_ showed no significant change, and the half-life and average residence time were also extended (*P* > 0.05). The AUC _(0-*t*)_ of PF in the compatibility group was increased (*P* < 0.01). Both C _max_ and AUC _(0-∞)_ were increased, and the half-life and average residence time were shortened (*P* > 0.05).

### 3.7. Changes of TP in Tissues before and after Compatibility

**Figure 2:**
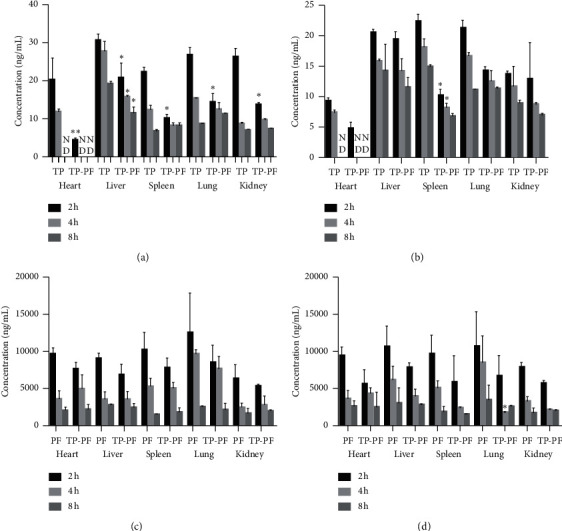
(a) Concentration changes of TP in heart, liver, spleen, lung, and kidney before and after TP combined with PF in male rats. (b) Concentration changes of TP in heart, liver, spleen, lung, and kidney before and after TP combined with PF in female rats. (c) Concentration changes of PF in heart, liver, spleen, lung, and kidney before and after TP combined with PF in male rats. (d) Concentration changes of PF in heart, liver, spleen, lung, and kidney before and after TP combined with PF in female rats. During the methodological investigation, we did a study on the distribution of blank tissue, and the results showed that the blank had no effect on triptolide and paeoniflorin. ^*∗*^*P* < 0.05, a significant difference from TP group; ^*∗∗*^*P* < 0.01, highly significant from TP group; ND, no detected.

**Figure 3:**
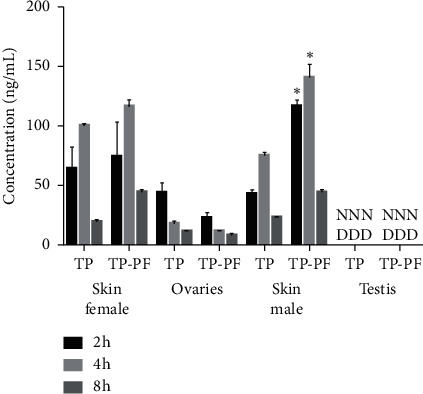
Concentration changes of TP in skin, ovaries, and testes before and after TP combined with PF in rats. ^*∗*^*P* < 0.05, a significant difference from TP group; ^*∗∗*^*P* < 0.01, highly significant from TP group.

During the methodological investigation, we did a study on the distribution of blank tissue, and the results showed that the blank had no effect on triptolide and paeoniflorin. After transdermal administration of the combination of TP and PF, the content of TP in the heart, liver, spleen, lungs, and kidneys of male rats was decreased at 2 h (*P* < 0.05), and the drug concentration in liver tissue was decreased at 2 h, 4 h, and 8 h (*P* < 0.05) (Figure 2(a)). The content of TP in the spleen of female rats was decreased at 2 h and 4 h (*P* < 0.05) and was decreased in other tissues as well (*P* > 0.05) (Figure 2(b)). There was no significant difference in the content of PF in the heart, liver, spleen, lungs, and kidneys of male and female rats after transdermal administration of the combination of TP and PF (*P* > 0.05) (Figures 2(c) and 2(d)). After transdermal administration of the combination of TP and PF, the concentration of TP in the skin increased (*P* < 0.05) (Figure 3).

## 4. Discussion

After the transdermal administration of the combination of TP and PF, there was no significant difference in the area under the blood concentration-time curve of TP, the peak concentration decreased, and the peak time was delayed, indicating that the absorption of TP in rats was slowed and delayed after the combined administration of the two drugs. The change of the drug-time curve also showed that TP had a more stable blood concentration change after compatibility. The above suggested that the compatibility of the two may weaken the toxicity of TP after percutaneous administration. *C*_max_ of PF increased by 2.38 times; AUC_(0-*t*)_, peak concentration and AUC_(0-∞)_ increased; and half-life and average retention time were shortened, suggesting that the combination of the two drugs can promote the transdermal absorption of PF after percutaneous administration and may enhance its efficacy [17]. This could be due to the fact that TP can cause swelling and ulceration of the skin of rats [9], reducing the barrier function of the skin, which may increase the absorption of PF. PF reduces the levels of skin inflammatory factors and relieves the symptoms of skin lesions [18], which may slow the absorption of TP.

After transdermal administration of TP and PF, the content of TP in the tissues of male rats decreased. This may be caused by the specific expression of CYP3A2 in male rats and the accelerated metabolism of TP [19]. However, after the two drugs were percutaneously administered, the concentration of TP in the skin increased, suggesting that the amount of TP retained in the skin increased, thereby reducing its content in the blood and tissues, which in turn may have caused an attenuation effect. It was found that the skin of male rats was thicker than that of female rats [20]. These study results showed that the content of TP in male rats skin was higher than that in female rats skin after compatibility at 2 h and 4 h, which may be related to the difference of male and female skin barrier. The ovaries of female rats in the TP group had higher concentrations of TP, suggesting that TP may have strong female reproductive organ toxicity [21]. It was found that the skin of male rats was thicker than that of female rats. This study results showed that the content of TP in male rats skin was higher than that in female rats skin after compatibility, which may be related to the difference of male and female skin barrier.

In conclusion, this is the first report to evaluate the pharmacokinetics and tissue distribution of the combination of TP and PF in rats after transdermal administration. The absorption of TP in rats was slowed and delayed. Additionally, the change of plasma concentration was more stable, and the absorption of PF in rats was accelerated. Furthermore, the TP content in the heart, spleen, lungs, and kidneys of male rats decreased significantly at 2 h. The drug concentration in liver tissue decreased significantly at 2 h, 4 h, and 8 h. However, there was no significant difference in the PF content in the heart, liver, spleen, lungs, and kidneys. In this report, we studied the pharmacological effects and tissue distribution of the combination of TP and PF after transdermal administration in male and female rats for the first time. This work will provide useful information for the clinical application and further research of TP.

## Figures and Tables

**Table 1:** Calibration curves for TP and PF in plasma.

Plasma samples	Linear ranges (ng·mL^−1^)	Calibration curves	Correlation coefficients
Triptolide	5–500	*Y* = 0.0036*X* − 0.0163	*r* ^2^ = 0.9949
Paeoniflorin	100–100000	*Y* = 0.0003*X* − 0.11	*r* ^2^ = 0.9928

**Table 2:** Regression equation and linearity range of TP in tissues.

Tissues	Linearity range (ng·mL^−1^)	Calibration curves	Correlation coefficients
Heart	5–500	*Y* = 0.011*X* + 0.0175	*r* ^2^ = 0.9979
Liver	5–500	*Y* = 0.0125*X* − 0.1381	*r* ^2^ = 0.9941
Spleen	5–500	*Y* = 0.0117*X* − 0.0565	*r* ^2^ = 0.9991
Lung	5–500	*Y* = 0.0125*X* − 0.0856	*r* ^2^ = 0.999
Kidney	5–500	*Y* = 0.0125*X* − 0.0631	*r* ^2^ = 0.9981
Skin	5–500	*Y* = 0.0142*X* − 0.1261	*r* ^2^ = 0.9985
Ovaries	5–500	*Y* = 0.0149*X* − 0.1673	*r* ^2^ = 0.9949
Testis	5–500	*Y* = 0.0119*X* − 0.1118	*r* ^2^ = 0.998

**Table 3:** Regression equation and linearity range of PF in tissues.

Tissues	Linearity range (ng·mL^−1^)	Calibration curves	Correlation coefficients
Heart	100–100000	*Y* = 0.00004*X* + 0.5649	*r* ^2^ = 0.998
Liver	100–100000	*Y* = 0.00005*X* + 0.5589	*r* ^2^ = 0.996
Spleen	100–100000	*Y* = 0.0001*X* + 0.649	*r* ^2^ = 0.997
Lung	100–100000	*Y* = 0.0001*X* + 0.466	*r* ^2^ = 0.997
Kidney	100–100000	*Y* = 0.00008*X* + 0.5319	*r* ^2^ = 0.993
Skin	100–100000	*Y* = 0.00006*X* + 0.5658	*r* ^2^ = 0.995
Ovaries	100–100000	*Y* = 0.00006*X* + 0.6135	*r* ^2^ = 0.998
Testis	100–100000	*Y* = 0.00006*X* + 0.6526	*r* ^2^ = 0.993

**Table 4:** Accuracy and precision of TP and PF in plasma (*n* = 6).

Analyses	Spiked concentration (ng·mL^−1^)	Intraday			Interday		
		Found concentration (mean ± SD)	Precision (RSD, %)	Accuracy (RE, %)	Found concentration (mean ± SD)	Precision (RSD, %)	Accuracy (RE, %)
Triptolide	15	13.25 ± 0.74	5.62	−11.66	14.01 ± 0.74	5.31	−6.59
	50	55.91 ± 6.64	11.87	11.82	56.36 ± 2.21	3.92%	12.71
	400	376.67 ± 37.69	10.01	−5.83	386.97 ± 37.69	9.74	−3.26

Paeoniflorin	300	302.48 ± 19.80	6.55	0.83	303.40 ± 22.21	7.32	1.13
	10000	10513.17 ± 1088.58	10.35	5.13	10488.17 ± 1066.05	10.16	4.88
	90000	89246.50 ± 9568.47	10.72	−0.84	87496.50 ± 10240.51	11.70	−2.78

**Table 5:** Accuracy and precision of TP in tissues (*n* = 5).

Tissue	Spiked (ng·mL^−1^)	Intraday			Interday		
		Concentration (mean ± SD)	Precision (RSD, %)	Accuracy (RE, %)	Concentration (mean ± SD)	Precision (RSD, %)	Accuracy (RE, %)
Heart	15	14.61 ± 1.21	8.31	−2.61	14.99 ± 1.35	8.98	−0.07
	40	38.50 ± 1.50	3.90	−3.74	38.92 ± 1.70	4.36	−2.71
	400	394.28 ± 11.34	2.88	−1.43	398.76 ± 30.32	7.60	−0.31

Liver	15	16.37 ± 0.08	0.48	9.14	16.18 ± 0.28	1.75	7.88
	40	41.00 ± 0.71	1.72	2.51	40.91 ± 0.59	1.43	2.28
	400	404.81 ± 9.48	2.34	1.20	385.93 ± 20.37	5.28	−3.52

Spleen	15	15.06 ± 0.36	2.40	0.41	15.04 ± 0.34	2.24	0.29
	40	36.01 ± 1.59	4.41	−9.97	35.51 ± 1.60	4.51	−11.22
	400	435.69 ± 2.71	0.62	8.92	424.61 ± 11.28	2.66	6.15

Lung	15	14.46 ± 0.12	0.82	−3.60	14.52 ± 0.11	0.77	−3.18
	40	42.65 ± 0.40	0.93	6.62	42.82 ± 0.49	1.13	7.06
	400	381.37 ± 6.12	1.60	−4.66	386.76 ± 7.15	1.85	−3.31

Kidney	15	14.15 ± 0.63	4.46	−5.67	14.33 ± 0.68	4.74	−4.48
	40	44.76 ± 2.61	5.83	11.90	44.57 ± 2.28	5.12	11.42
	400	422.50 ± 17.61	4.17	5.63	414.30 ± 18.52	4.47	3.57

Skin	15	16.67 ± 0.23	1.39	11.14	16.73 ± 0.22	1.31	11.56
	40	43.19 ± 1.12	2.59	7.98	43.63 ± 1.81	4.15	9.09
	400	436.20 ± 10.90	2.50	9.05	441.32 ± 16.55	3.75	10.33

Ovaries	15	15.97 ± 0.48	2.98	6.48	15.99 ± 0.68	4.27	6.58
	40	44.05 ± 2.86	6.49	10.12	45.08 ± 3.34	7.40	12.69
	400	440.01 ± 30.11	6.84	10.00	442.19 ± 28.03	6.34	10.55

Testis	15	16.25 ± 0.42	2.60	8.32	16.34 ± 0.22	1.35	8.91
	40	44.07 ± 1.05	2.38	10.17	44.39 ± 1.20	2.71	10.98
	400	387.58 ± 10.05	2.98	−3.11	385.66 ± 4.35	1.13%	−3.59

**Table 6:** Accuracy and precision of PF in tissues (*n* = 5).

Tissue	Spiked (ng·mL^−1^)	Intraday			Interday		
		Concentration (mean ± SD)	Precision (RSD, %)	Accuracy (RE, %)	Concentration (mean ± SD)	Precision (RSD, %)	Accuracy (RE, %)
Heart	300	343.42 ± 12.14	3.53	14.47	327.72 ± 21.19	6.47	9.24
	10000	9644.25 ± 304.18	3.15	−3.56	9541.73 ± 361.92	3.79	−4.58
	90000	96372.10 ± 8760.18	9.09	7.08	98474.31 ± 13288.96	13.49	9.42

Liver	300	335.11 ± 38.61	11.52	11.70	337.11 ± 28.16	8.35	12.37
	10000	9586.85 ± 283.46	2.96	−4.13	8970.58 ± 980.56	10.93	−10.29
	90000	87937.55 ± 4493.29	5.11	−2.29	87986.22 ± 3833.75	4.36	−2.24

Spleen	300	308.90 ± 44.73	14.48	2.97	314.58 ± 41.18	13.09	4.86
	10000	9557.40 ± 306.28	3.20	−4.43	9606.79 ± 377.57	3.93	−3.93
	90000	94573.89 ± 11563.05	12.23	5.08	91691.47 ± 12416.90	13.54	1.88

Lung	300	275.21 ± 31.98	9.77	−8.26	273.96 ± 23.21	8.47	−4.62
	10000	8870.60 ± 1009.65	11.38	−11.29	9099.30 ± 957.97	10.53	−9.01
	90000	86423.35 ± 8446.97	11.62	−3.97	85838.68 ± 8825.90	10.28	−8.68

Kidney	300	319.14 ± 21.46	6.72	6.38	314.52 ± 30.42	9.67	4.84
	10000	8674.69 ± 538.08	6.20	−13.25	8895.32 ± 618.98	6.96	−11.05
	90000	88127.48 ± 4606.73	5.23	−2.08	84696.41 ± 5361.51	6.33	−5.89

Skin	300	307.29 ± 12.17	3.96	2.43	317.74 ± 17.37	5.47	5.91
	10000	8811.53 ± 498.49	5.66	−11.88	9125.19 ± 553.29	6.06	−8.75
	90000	94989.87 ± 3753.00	3.95	5.54	94842.02 ± 2849.43	3.00	5.38

Ovaries	300	319.54 ± 19.73	6.17	6.51	309.61 ± 33.47	10.81	3.20
	10000	9129.07 ± 475.45	5.21	−8.71	8844.32 ± 965.24	10.91	−11.56
	90000	93800.86 ± 5510.22	5.87	4.22	94747.39 ± 12999.54	13.72	5.27

Testis	300	303.83 ± 30.03	9.89	1.28	308.22 ± 24.09	7.82	2.74
	10000	9143.88 ± 305.43	3.34	−8.56	9379.53 ± 530.38	5.65	−6.20
	90000	95800.87 ± 7372.54	7.70	6.45	93591.79 ± 8891.62	9.50	3.99

**Table 7:** Main pharmacokinetic parameters of TP and PF in rats.

Parameters	Triptolide		Paeoniflorin	
	TP	TP-PF	PF	TP-PF
*C*_max_ (*μ*g/L)	63.42 ± 17.40	36.89 ± 12.73	22383.66 ± 7768.60	53314.70 ± 19958.50
*T*_max_ (h)	0.88 ± 0.23	2.25 ± 1.23	0.75 ± 0.29	2.08 ± 2.11
*t*1/2*z* (h)	26.18 ± 17.22	59.15 ± 63.89	37.27 ± 12.70	16.37 ± 8.05
AUC (0-*t*) (*μ*g/L^*∗*^h)	247.95 ± 54.35	289.57 ± 37.30	113701.69 ± 7470.15	214379.35 ± 24618.58^*∗∗*^
AUC (0-∞) (*μ*g/L^*∗*^h)	569.22 ± 264.16	987.70 ± 728.02^*∗∗*^	287530.42 ± 98387.10	337060.99 ± 120757.76
MRT (0-*t*) (h)	7.56 ± 1.22	9.42 ± 0.84	8.58 ± 0.71	7.58 ± 1.36
MRT (0-∞) (h)	30.85 ± 22.54	82.67 ± 90.21	50.01 ± 19.60	22.86 ± 12.40
CLz/F (L/h/kg)	2.01 ± 0.74	1.35 ± 0.63	3.78 ± 1.22	3.25 ± 1.07
Vz/f (L/kg)	82.04 ± 51.74	73.98 ± 23.85	68.22 ± 18.27	187.44 ± 5.28

Compared with the monodrug group, ^*∗*^*P* < 0.05 and ^*∗∗*^*P* < 0.01.

## Data Availability

All datasets presented in this study are included within the article/supplementary material.

## Supplementary Materials

Supplementary Materials Figure S1: chromatograms of plasma. (A) Blank plasma sample of TP group; (B) blank spiked with TP (I) and carbamazepine (II); (C) samples after 30 min of administration TP (I) and IS (II), respectively. (D) Blank plasma sample of PF group; (E) blank spiked with PF (I) and carbamazepine (II); (F) samples after 30 min of administration PF (I) and carbamazepine (II), respectively. Figure S2. Chromatograms of typical tissues. (A) Blank tissues sample of TP group; (B) blank spiked with TP (I) and carbamazepine (II); (C) samples after 30 min of administration of TP (I) and carbamazepine (II), respectively. (D) Blank tissues sample of PF group (E) blank spiked with PF (I) and carbamazepine (II); (F) samples after 30 min of administration of PF(I) and carbamazepine (II), respectively. Table S1: recovery and matrix effect for the analysis of TP and PF in plasma (*n* = 6). Table S2: recovery and matrix effect of TP in tissues (*n* = 5). Table S3: recovery and matrix effect of PF in tissues (*n* = 5). Table S4: stability of TP in plasma (*n* = 6). Table S5: stability of PF in plasma (*n* = 6). Table S6: stability of TP in tissues. Table S7: stability of PF in tissues.
