# Supplementary material for: Pharmacokinetics and Tissue Distribution of Combined Triptolide and Paeoniflorin Regimen for Percutaneous Administration in Rats Assessed by Liquid Chromatography-Tandem Mass Spectrometry
Source: Evid Based Complement Alternat Med. 2021 Jul 8;2021:8864273. doi: 10.1155/2021/8864273 (PMC8282371; doi:10.1155/2021/8864273)
Supplement: Supplementary Materials — Figure S1: chromatograms of plasma. (A) Blank plasma sample of TP group; (B) blank spiked with TP (I) and carbamazepine (II); (C) samples after 30 min of administration TP (I) and IS (II), respectively. (D) Blank plasma sample of PF group; (E) blank spiked with PF (I) and carbamazepine (II); (F) samples after 30 min of administration PF (I) and carbamazepine (II), respectively. Figure S2. Chromatograms of typical tissues. (A) Blank tissues sample of TP group; (B) blank spiked with TP (I) and carbamazepine (II); (C) samples after 30 min of administration of TP (I) and carbamazepine (II), respectively. (D) Blank tissues sample of PF group (E) blank spiked with PF (I) and carbamazepine (II); (F) samples after 30 min of administration of PF(I) and carbamazepine (II), respectively. Table S1: recovery and matrix effect for the analysis of TP and PF in plasma (n = 6). Table S2: recovery and matrix effect of TP in tissues (n = 5). Table S3: recovery and matrix effect of PF in tissues (n = 5). Table S4: stability of TP in plasma (n = 6). Table S5: stability of PF in plasma (n = 6). Table S6: stability of TP in tissues. Table S7: stability of PF in tissues. [file 8864273.f1.zip › 8864273.f1/Figure S1,S2 label.pdf]

Figure S1. Chromatograms of plasma. (A) blank plasma sample of TP group (B) blank spiked with TP (I) and carbamazepine (II) (C) samples after 30min of administration TP (I) and IS (II), respectively. (D) blank plasma sample of PF group (E) blank spiked with PF (I) and carbamazepine (II) (F) samples after 30min of administration PF (I) and carbamazepine (II), respectively.

Figure S2. Chromatograms of typical tissues. (A) blank tissues sample of TP group (B) blank spiked with TP (I) and carbamazepine (II) (C) samples after 30min of administration of TP (I) and carbamazepine (II), respectively. (D) blank tissues sample of PF group (E) blank spiked with PF (I) and carbamazepine (II) (F) samples after 30min of administration of PF(I) and carbamazepine (II), respectively.
