# Supplementary material for: Pharmacokinetics and Tissue Distribution of Combined Triptolide and Paeoniflorin Regimen for Percutaneous Administration in Rats Assessed by Liquid Chromatography-Tandem Mass Spectrometry
Source: Evid Based Complement Alternat Med. 2021 Jul 8;2021:8864273. doi: 10.1155/2021/8864273 (PMC8282371; doi:10.1155/2021/8864273)
Supplement: Supplementary Materials — Figure S1: chromatograms of plasma. (A) Blank plasma sample of TP group; (B) blank spiked with TP (I) and carbamazepine (II); (C) samples after 30 min of administration TP (I) and IS (II), respectively. (D) Blank plasma sample of PF group; (E) blank spiked with PF (I) and carbamazepine (II); (F) samples after 30 min of administration PF (I) and carbamazepine (II), respectively. Figure S2. Chromatograms of typical tissues. (A) Blank tissues sample of TP group; (B) blank spiked with TP (I) and carbamazepine (II); (C) samples after 30 min of administration of TP (I) and carbamazepine (II), respectively. (D) Blank tissues sample of PF group (E) blank spiked with PF (I) and carbamazepine (II); (F) samples after 30 min of administration of PF(I) and carbamazepine (II), respectively. Table S1: recovery and matrix effect for the analysis of TP and PF in plasma (n = 6). Table S2: recovery and matrix effect of TP in tissues (n = 5). Table S3: recovery and matrix effect of PF in tissues (n = 5). Table S4: stability of TP in plasma (n = 6). Table S5: stability of PF in plasma (n = 6). Table S6: stability of TP in tissues. Table S7: stability of PF in tissues. [file 8864273.f1.zip › 8864273.f1/Table S1 (1).docx]

Table S1 Recovery and matrix effect for the analysis of TP and PF in plasma (n=6)

| Analyzes | Spiked concentration  (ng·mL^-1^) | Recovery (%) | RSD (%) | The matrix effect (%) | RSD (%) |
| --- | --- | --- | --- | --- | --- |
| Triptolide | 15 | 100.81 | 12.78 | 114.21 | 7.90 |
|  | 50 | 96.80 | 10.71 | 110.47 | 13.01 |
|  | 400 | 73.22 | 2.98 | 89.61 | 2.43 |
| Paeoniflorin | 300 | 45.97 | 8.45 | 105.10 | 6.61 |
|  | 10 000 | 38.17 | 12.14 | 117.64 | 4.02 |
|  | 90 000 | 45.73 | 14.28 | 103.32 | 5.33 |
