# Supplementary material for: Pharmacokinetics and Tissue Distribution of Combined Triptolide and Paeoniflorin Regimen for Percutaneous Administration in Rats Assessed by Liquid Chromatography-Tandem Mass Spectrometry
Source: Evid Based Complement Alternat Med. 2021 Jul 8;2021:8864273. doi: 10.1155/2021/8864273 (PMC8282371; doi:10.1155/2021/8864273)
Supplement: Supplementary Materials — Figure S1: chromatograms of plasma. (A) Blank plasma sample of TP group; (B) blank spiked with TP (I) and carbamazepine (II); (C) samples after 30 min of administration TP (I) and IS (II), respectively. (D) Blank plasma sample of PF group; (E) blank spiked with PF (I) and carbamazepine (II); (F) samples after 30 min of administration PF (I) and carbamazepine (II), respectively. Figure S2. Chromatograms of typical tissues. (A) Blank tissues sample of TP group; (B) blank spiked with TP (I) and carbamazepine (II); (C) samples after 30 min of administration of TP (I) and carbamazepine (II), respectively. (D) Blank tissues sample of PF group (E) blank spiked with PF (I) and carbamazepine (II); (F) samples after 30 min of administration of PF(I) and carbamazepine (II), respectively. Table S1: recovery and matrix effect for the analysis of TP and PF in plasma (n = 6). Table S2: recovery and matrix effect of TP in tissues (n = 5). Table S3: recovery and matrix effect of PF in tissues (n = 5). Table S4: stability of TP in plasma (n = 6). Table S5: stability of PF in plasma (n = 6). Table S6: stability of TP in tissues. Table S7: stability of PF in tissues. [file 8864273.f1.zip › 8864273.f1/Table S2 (1).docx]

Table S2 Recovery and matrix effect of TP in tissues (n=5)

| Tissues | Spiked concentration  (ng·mL^-1^) | Recovery(%) | RSD(%) | The matrix effect(%) | RSD(%) |
| --- | --- | --- | --- | --- | --- |
| Heart | 15 | 97.87 | 8.86 | 98.59 | 2.50 |
|  | 40 | 83.78 | 8.48 | 96.32 | 0.98 |
|  | 400 | 82.24 | 4.05 | 91.36 | 7.50 |
| Liver | 15 | 99.78 | 2.82 | 102.88 | 2.92 |
|  | 40 | 98.84 | 2.29 | 102.30 | 1.52 |
|  | 400 | 94.08 | 8.14 | 105.56 | 4.97 |
| Spleen | 15 | 76.42 | 5.30 | 112.39 | 9.65 |
|  | 40 | 72.16 | 4.58 | 101.55 | 6.82 |
|  | 400 | 73.22 | 7.38 | 98.14 | 0.96 |
| Lung | 15 | 82.41 | 3.80 | 107.40 | 4.12 |
|  | 40 | 86.36 | 11.01 | 86.26 | 10.56 |
|  | 400 | 79.31 | 6.32 | 94.75 | 11.87 |
| Kidney | 15 | 101.74 | 3.02 | 110.04 | 0.84 |
|  | 40 | 91.01 | 4.49 | 111.08 | 3.59 |
|  | 400 | 101.93 | 2.83 | 98.95 | 7.99 |
| Skin | 15 | 97.17 | 1.63 | 102.25 | 1.97 |
|  | 40 | 96.47 | 12.53 | 97.99 | 2.00 |
|  | 400 | 97.74 | 0.08 | 97.27 | 4.26 |
| Ovaries | 15 | 97.47 | 13.26 | 103.28 | 8.43 |
|  | 40 | 95.71 | 3.71 | 108.89 | 3.01 |
|  | 400 | 99.51 | 10.65 | 99.48 | 5.00 |
| Testis | 15 | 98.01 | 2.33 | 100.57 | 3.50 |
|  | 40 | 97.63 | 2.64 | 103.27 | 3.24 |
|  | 400 | 94.13 | 8.41 | 100.57 | 3.45 |
