# Supplementary material for: Pharmacokinetics and Tissue Distribution of Combined Triptolide and Paeoniflorin Regimen for Percutaneous Administration in Rats Assessed by Liquid Chromatography-Tandem Mass Spectrometry
Source: Evid Based Complement Alternat Med. 2021 Jul 8;2021:8864273. doi: 10.1155/2021/8864273 (PMC8282371; doi:10.1155/2021/8864273)
Supplement: Supplementary Materials — Figure S1: chromatograms of plasma. (A) Blank plasma sample of TP group; (B) blank spiked with TP (I) and carbamazepine (II); (C) samples after 30 min of administration TP (I) and IS (II), respectively. (D) Blank plasma sample of PF group; (E) blank spiked with PF (I) and carbamazepine (II); (F) samples after 30 min of administration PF (I) and carbamazepine (II), respectively. Figure S2. Chromatograms of typical tissues. (A) Blank tissues sample of TP group; (B) blank spiked with TP (I) and carbamazepine (II); (C) samples after 30 min of administration of TP (I) and carbamazepine (II), respectively. (D) Blank tissues sample of PF group (E) blank spiked with PF (I) and carbamazepine (II); (F) samples after 30 min of administration of PF(I) and carbamazepine (II), respectively. Table S1: recovery and matrix effect for the analysis of TP and PF in plasma (n = 6). Table S2: recovery and matrix effect of TP in tissues (n = 5). Table S3: recovery and matrix effect of PF in tissues (n = 5). Table S4: stability of TP in plasma (n = 6). Table S5: stability of PF in plasma (n = 6). Table S6: stability of TP in tissues. Table S7: stability of PF in tissues. [file 8864273.f1.zip › 8864273.f1/Table S3 (1).docx]

Table S3 Recovery and matrix effect of PF in tissues (n=5)

| Tissues | Spiked concentration  (ng·mL^-1^) | Recovery(%) | RSD(%) | The matrix effect(%) | RSD(%) |
| --- | --- | --- | --- | --- | --- |
| Heart | 300 | 44.63 | 3.45 | 96.74 | 10.02 |
|  | 10 000 | 55.48 | 2.76 | 105.19 | 12.54 |
|  | 90 000 | 46.51 | 11.84 | 98.09 | 14.05 |
| Liver | 300 | 61.78 | 13.73 | 90.40 | 9.28 |
|  | 10 000 | 44.99 | 10.75 | 106.65 | 13.07 |
|  | 90 000 | 54.25 | 4.37 | 99.59 | 9.72 |
| Spleen | 300 | 56.56 | 11.80 | 94.94 | 12.30 |
|  | 10 000 | 41.21 | 8.07 | 105.81 | 7.05 |
|  | 90 000 | 42.64 | 8.68 | 90.06 | 9.18 |
| Lung | 300 | 48.17 | 7.14 | 102.94 | 6.01 |
|  | 10 000 | 51.21 | 8.49 | 97.31 | 2.07 |
|  | 90 000 | 41.39 | 1.38 | 90.21 | 13.82 |
| Kidney | 300 | 46.77 | 4.37 | 97.69 | 12.03 |
|  | 10 000 | 45.24 | 11.77 | 103.36 | 14.93 |
|  | 90 000 | 34.35 | 8.59 | 99.63 | 8.70 |
| Skin | 300 | 48.36 | 10.73 | 98.87 | 4.37 |
|  | 10 000 | 40.37 | 6.79 | 98.28 | 10.94 |
|  | 90 000 | 36.87 | 12.99 | 95.91 | 4.81 |
| Ovaries | 300 | 64.30 | 13.19 | 100.68 | 11.78 |
|  | 10 000 | 59.11 | 4.07 | 92.56 | 3.89 |
|  | 90 000 | 54.03 | 2.90 | 97.54 | 5.41 |
| Testis | 300 | 61.05 | 6.53 | 99.24 | 3.21 |
|  | 10 000 | 40.99 | 5.42 | 95.40 | 14.10 |
|  | 90 000 | 40.89 | 10.93 | 95.93 | 14.50 |
