# Supplementary material for: Pharmacokinetics and Tissue Distribution of Combined Triptolide and Paeoniflorin Regimen for Percutaneous Administration in Rats Assessed by Liquid Chromatography-Tandem Mass Spectrometry
Source: Evid Based Complement Alternat Med. 2021 Jul 8;2021:8864273. doi: 10.1155/2021/8864273 (PMC8282371; doi:10.1155/2021/8864273)
Supplement: Supplementary Materials — Figure S1: chromatograms of plasma. (A) Blank plasma sample of TP group; (B) blank spiked with TP (I) and carbamazepine (II); (C) samples after 30 min of administration TP (I) and IS (II), respectively. (D) Blank plasma sample of PF group; (E) blank spiked with PF (I) and carbamazepine (II); (F) samples after 30 min of administration PF (I) and carbamazepine (II), respectively. Figure S2. Chromatograms of typical tissues. (A) Blank tissues sample of TP group; (B) blank spiked with TP (I) and carbamazepine (II); (C) samples after 30 min of administration of TP (I) and carbamazepine (II), respectively. (D) Blank tissues sample of PF group (E) blank spiked with PF (I) and carbamazepine (II); (F) samples after 30 min of administration of PF(I) and carbamazepine (II), respectively. Table S1: recovery and matrix effect for the analysis of TP and PF in plasma (n = 6). Table S2: recovery and matrix effect of TP in tissues (n = 5). Table S3: recovery and matrix effect of PF in tissues (n = 5). Table S4: stability of TP in plasma (n = 6). Table S5: stability of PF in plasma (n = 6). Table S6: stability of TP in tissues. Table S7: stability of PF in tissues. [file 8864273.f1.zip › 8864273.f1/Table S4 (1).docx]

Table S4 Stability of TP in plasma (n=6)

| Storge conditions | Spiked  Concentration  (ng·mL^-1^) | Found  Concentration  (ng·mL^-1^) | RSD  (%) | RE  (%) |
| --- | --- | --- | --- | --- |
| Short-term  (6h,room temperature) | 15 | 14.77 ±0.69 | 4.70 | -1.53 |
|  | 50 | 56.33 ±2.58 | 4.59 | 12.66 |
|  | 400 | 445.19 ±38.09 | 8.56 | 11.30 |
| Long-term  (30 days, -20℃ ) | 15 | 15.87 ±0.77 | 4.85 | 5.78 |
|  | 50 | 50.78 ±3.25 | 6.39 | 1.56 |
|  | 400 | 371.76 ±54.17 | 14.57 | -7.06 |
| Three freeze-thaw  cycles (-20℃) | 15 | 14.13 ±0.51 | 3.58 | -5.78 |
|  | 50 | 57.37 ±4.08 | 7.11 | 14.75 |
|  | 400 | 418.17 ±54.19 | 12.96 | 4.54 |
| Autosampler  Stability at 4℃  for 20h | 15 | 14.29 ±0.75 | 5.25 | -4.76 |
|  | 50 | 53.01 ±0.96 | 1.82 | 6.01 |
|  | 400 | 436.63 ±37.86 | 8.67 | 9.16 |
