# Supplementary material for: Pharmacokinetics and Tissue Distribution of Combined Triptolide and Paeoniflorin Regimen for Percutaneous Administration in Rats Assessed by Liquid Chromatography-Tandem Mass Spectrometry
Source: Evid Based Complement Alternat Med. 2021 Jul 8;2021:8864273. doi: 10.1155/2021/8864273 (PMC8282371; doi:10.1155/2021/8864273)
Supplement: Supplementary Materials — Figure S1: chromatograms of plasma. (A) Blank plasma sample of TP group; (B) blank spiked with TP (I) and carbamazepine (II); (C) samples after 30 min of administration TP (I) and IS (II), respectively. (D) Blank plasma sample of PF group; (E) blank spiked with PF (I) and carbamazepine (II); (F) samples after 30 min of administration PF (I) and carbamazepine (II), respectively. Figure S2. Chromatograms of typical tissues. (A) Blank tissues sample of TP group; (B) blank spiked with TP (I) and carbamazepine (II); (C) samples after 30 min of administration of TP (I) and carbamazepine (II), respectively. (D) Blank tissues sample of PF group (E) blank spiked with PF (I) and carbamazepine (II); (F) samples after 30 min of administration of PF(I) and carbamazepine (II), respectively. Table S1: recovery and matrix effect for the analysis of TP and PF in plasma (n = 6). Table S2: recovery and matrix effect of TP in tissues (n = 5). Table S3: recovery and matrix effect of PF in tissues (n = 5). Table S4: stability of TP in plasma (n = 6). Table S5: stability of PF in plasma (n = 6). Table S6: stability of TP in tissues. Table S7: stability of PF in tissues. [file 8864273.f1.zip › 8864273.f1/Table S5 (1).docx]

Table S5 Stability of PF in plasma (n=6)

| Storge conditions | Spiked  Concentration  (ng·mL^-1^) | Found  Concentration  (ng·mL^-1^) | RSD  (%) | RE  (%) |
| --- | --- | --- | --- | --- |
| Short-term  (6h,room temperature) | 300 | 304.32 ±24.36 | 8.00 | 1.44 |
|  | 10 000 | 10463.17±1042.43 | 9.96 | 4.63 |
|  | 90 000 | 85746.50±10585.63 | 12.35 | -4.73 |
| Long-term  (30 days, -20℃ ) | 300 | 301.65 ±22.88 | 7.59 | 0.55 |
|  | 10 000 | 10429.84 ±1126.45 | 10.80 | 4.30 |
|  | 90 000 | 85413.17±9600.35 | 11.24 | -5.10 |
| Three freeze-thaw  cycles (-20℃) | 300 | 306.15 ±23.92 | 7.81 | 2.05 |
|  | 10 000 | 10546.50 ±1200.23 | 11.38 | 5.47 |
|  | 90 000 | 85413.17 ±7788.88 | 9.12 | -5.10 |
| Autosampler  Stability at 4℃for 20h | 300 | 345.98 ±32.01 | 9.25 | 15.33 |
|  | 10 000 | 10913.17 ± 884.12 | 8.10 | 9.13 |
|  | 90 000 | 94913.17±10637.98 | 11.21 | 5.46 |
