# Supplementary material for: Pharmacokinetics and Tissue Distribution of Combined Triptolide and Paeoniflorin Regimen for Percutaneous Administration in Rats Assessed by Liquid Chromatography-Tandem Mass Spectrometry
Source: Evid Based Complement Alternat Med. 2021 Jul 8;2021:8864273. doi: 10.1155/2021/8864273 (PMC8282371; doi:10.1155/2021/8864273)
Supplement: Supplementary Materials — Figure S1: chromatograms of plasma. (A) Blank plasma sample of TP group; (B) blank spiked with TP (I) and carbamazepine (II); (C) samples after 30 min of administration TP (I) and IS (II), respectively. (D) Blank plasma sample of PF group; (E) blank spiked with PF (I) and carbamazepine (II); (F) samples after 30 min of administration PF (I) and carbamazepine (II), respectively. Figure S2. Chromatograms of typical tissues. (A) Blank tissues sample of TP group; (B) blank spiked with TP (I) and carbamazepine (II); (C) samples after 30 min of administration of TP (I) and carbamazepine (II), respectively. (D) Blank tissues sample of PF group (E) blank spiked with PF (I) and carbamazepine (II); (F) samples after 30 min of administration of PF(I) and carbamazepine (II), respectively. Table S1: recovery and matrix effect for the analysis of TP and PF in plasma (n = 6). Table S2: recovery and matrix effect of TP in tissues (n = 5). Table S3: recovery and matrix effect of PF in tissues (n = 5). Table S4: stability of TP in plasma (n = 6). Table S5: stability of PF in plasma (n = 6). Table S6: stability of TP in tissues. Table S7: stability of PF in tissues. [file 8864273.f1.zip › 8864273.f1/Table S6 (1).docx]

Table S6 Stability of TP in tissues

| Tissues | Spiked  (ng·mL^-1^) | Stability a | | Stability b | | Stability c | |
| --- | --- | --- | --- | --- | --- | --- | --- |
|  |  | Measured  (ng·mL^-1^) | RSD  (%) | Measured  (ng·mL^-1^) | RSD  (%) | Measured  (ng·mL^-1^) | RSD  (%) |
| Heart | 15 | 15.41±1.06 | 6.90 | 15.43±1.19 | 7.69 | 15.37±1.37 | 8.88 |
|  | 40 | 38.96±1.86 | 4.76 | 38.64±1.95 | 5.05 | 39.33±1.78 | 4.52 |
|  | 400 | 400.86±32.81 | 8.18 | 398.53±36.31 | 9.11 | 403.23±40.86 | 10.13 |
| Liver | 15 | 16.12±0.28 | 1.71 | 16.08±0.29 | 1.83 | 15.99±0.29 | 1.81 |
|  | 40 | 40.71±0.40 | 0.98 | 40.67±0.44 | 1.08 | 40.82±0.41 | 1.01 |
|  | 400 | 379.83±16.58 | 4.37 | 373.59±12.20 | 3.26 | 367.05±5.24 | 1.43 |
| Spleen | 15 | 14.96±0.31 | 2.08 | 15.06±0.28 | 1.83 | 15.03±0.31 | 2.07 |
|  | 40 | 35.18±1.56 | 4.43 | 34.70±1.37 | 3.94 | 35.01±1.45 | 4.14 |
|  | 400 | 423.04±11.75 | 2.78 | 419.91±11.12 | 2.65 | 413.53±1.30 | 0.31 |
| Lung | 15 | 14.54±0.11 | 0.77 | 14.60±0.05 | 0.32 | 14.59±0.05 | 0.35 |
|  | 40 | 42.89±0.51 | 1.19 | 43.05±0.44 | 1.03 | 43.00±0.50 | 1.17 |
|  | 400 | 389.56±3.74 | 0.96 | 390.49±3.63 | 0.93 | 392.14±2.59 | 0.66 |
| Kidney | 15 | 14.38±0.73 | 5.11 | 14.23±0.76 | 5.31 | 14.51±0.68 | 4.67 |
|  | 40 | 44.50±2.49 | 5.61 | 45.25±2.23 | 4.92 | 44.38±1.88 | 4.24 |
|  | 400 | 408.72±14.99 | 3.67 | 411.03±15.94 | 3.88 | 406.10±15.53 | 3.82 |
| Skin | 15 | 16.70±0.22 | 1.30 | 16.77±0.19 | 1.15 | 16.82±0.20 | 1.17 |
|  | 40 | 44.04±1.72 | 3.90 | 44.08±1.92 | 4.35 | 44.07±2.22 | 5.03 |
|  | 400 | 439.82±17.75 | 4.04 | 440.40±19.80 | 4.50 | 446.44±19.40 | 4.35 |
| Ovaries | 15 | 16.19±0.56 | 3.48 | 16.24±0.62 | 3.81 | 15.97±0.48 | 2.98 |
|  | 40 | 44.16±2.89 | 6.53 | 44.85±2.84 | 6.32 | 44.05±2.86 | 6.49 |
|  | 400 | 444.71±30.07 | 6.76 | 450.10±31.39 | 6.97 | 440.01±30.11 | 6.84 |
| Testis | 15 | 16.37±0.23 | 1.40 | 16.42±0.23 | 1.38 | 16.33±0.18 | 1.09 |
|  | 40 | 44.11±1.13 | 2.56 | 44.45±1.01 | 2.27 | 44.16±1.01 | 2.29 |
|  | 400 | 337.41±2.05 | 0.61 | 337.40±2.29 | 0.68 | 336.91±2.46 | 0.73 |
