# Supplementary material for: Pharmacokinetics and Tissue Distribution of Combined Triptolide and Paeoniflorin Regimen for Percutaneous Administration in Rats Assessed by Liquid Chromatography-Tandem Mass Spectrometry
Source: Evid Based Complement Alternat Med. 2021 Jul 8;2021:8864273. doi: 10.1155/2021/8864273 (PMC8282371; doi:10.1155/2021/8864273)
Supplement: Supplementary Materials — Figure S1: chromatograms of plasma. (A) Blank plasma sample of TP group; (B) blank spiked with TP (I) and carbamazepine (II); (C) samples after 30 min of administration TP (I) and IS (II), respectively. (D) Blank plasma sample of PF group; (E) blank spiked with PF (I) and carbamazepine (II); (F) samples after 30 min of administration PF (I) and carbamazepine (II), respectively. Figure S2. Chromatograms of typical tissues. (A) Blank tissues sample of TP group; (B) blank spiked with TP (I) and carbamazepine (II); (C) samples after 30 min of administration of TP (I) and carbamazepine (II), respectively. (D) Blank tissues sample of PF group (E) blank spiked with PF (I) and carbamazepine (II); (F) samples after 30 min of administration of PF(I) and carbamazepine (II), respectively. Table S1: recovery and matrix effect for the analysis of TP and PF in plasma (n = 6). Table S2: recovery and matrix effect of TP in tissues (n = 5). Table S3: recovery and matrix effect of PF in tissues (n = 5). Table S4: stability of TP in plasma (n = 6). Table S5: stability of PF in plasma (n = 6). Table S6: stability of TP in tissues. Table S7: stability of PF in tissues. [file 8864273.f1.zip › 8864273.f1/Table S7 (1).docx]

Table S7 Stability of PF in tissues

| Tissues | Spiked  (ng·mL^-1^) | Stability a | | Stability b | | Stability c | |
| --- | --- | --- | --- | --- | --- | --- | --- |
|  |  | Measured  (ng·mL^-1^) | RSD  (%) | Measured  (ng·mL^-1^) | RSD  (%) | Measured  (ng·mL^-1^) | RSD  (%) |
| Heart | 300 | 323.43±14.00 | 4.33 | 322.66±23.08 | 7.15 | 312.03±16.07 | 5.15 |
|  | 10 000 | 9508.66±368.54 | 3.88 | 9574.75±407.97 | 4.26 | 9439.21±385.27 | 4.08 |
|  | 90 000 | 97656.72±9293.69 | 9.52 | 97090.79±8214.84 | 8.46 | 95264.14±8753.95 | 9.19 |
| Liver | 300 | 329.93±17.82 | 5.40 | 329.93±17.82 | 5.40 | 339.11±9.30 | 2.74 |
|  | 10 000 | 8842.74±1027.49 | 11.62 | 8780.30±967.75 | 11.02 | 8780.30±967.75 | 11.02 |
|  | 90 000 | 87303.04±3851.86 | 4.41 | 88034.89±3033.31 | 3.45 | 88034.89±3033.31 | 3.45 |
| Spleen | 300 | 328.36±29.92 | 9.11 | 325.02±32.60 | 10.03 | 342.53±13.86 | 4.05 |
|  | 10 000 | 9567.68±402.36 | 4.21 | 9678.21±375.87 | 3.88 | 9656.18±431.78 | 4.47 |
|  | 90 000 | 92297.11±13520.89 | 14.65 | 87688.01±11058.87 | 12.61 | 89964.75±11930.24 | 13.26 |
| Lung | 300 | 266.55±17.80 | 6.68 | 274.84±7.22 | 2.63 | 272.72±7.18 | 2.63 |
|  | 10 000 | 9073.14±1047.44 | 11.54 | 9467.90±769.59 | 8.13 | 9328.00±843.45 | 9.04 |
|  | 90 000 | 88110.53±7906.20 | 8.97 | 86993.44±8479.12 | 9.75 | 85254.00±9151.94 | 10.73 |
| Kidney | 300 | 307.70±28.83 | 9.37 | 310.06±31.80 | 10.26 | 309.90±36.72 | 11.85 |
|  | 10 000 | 9091.16±479.21 | 5.27 | 9087.46±535.71 | 5.89 | 9115.95±615.95 | 6.76 |
|  | 90 000 | 83257.02±4697.23 | 5.64 | 81359.14±3093.58 | 3.80 | 81265.33±3567.23 | 4.39 |
| Skin | 300 | 316.47±18.77 | 5.93 | 320.06±19.38 | 6.06 | 328.19±15.39 | 4.69 |
|  | 10 000 | 9272.68±486.67 | 5.25 | 9213.02±527.51 | 5.73 | 9438.85±408.66 | 4.33 |
|  | 90 000 | 95703.05±2301.06 | 2.40 | 94699.43±1257.94 | 1.33 | 94694.16±1452.50 | 1.53 |
| Ovaries | 300 | 306.01±35.59 | 11.63 | 309.42±39.05 | 12.62 | 299.68±40.67 | 13.57 |
|  | 10 000 | 8692.27±989.61 | 11.38 | 8701.94±1106.21 | 12.71 | 9201.62±795.52 | 8.65 |
|  | 90 000 | 93746.11±14027.49 | 14.96 | 93351.21±4835.13 | 5.18 | 92741.83±5448.48 | 5.87 |
| Testis | 300 | 306.70±26.13 | 8.52 | 300.09±25.20 | 8.40 | 312.61±14.84 | 4.75 |
|  | 10 000 | 9342.10±573.73 | 6.14 | 9425.37±613.82 | 6.51 | 9615.18±598.55 | 6.23 |
|  | 90 000 | 92813.08±9551.69 | 10.29 | 94504.41±9987.02 | 10.57 | 91382.71±9695.74 | 10.61 |
